# Synthesis and Antimicrobial Evaluation of 1-[(2-Substituted phenyl)carbamoyl]naphthalen-2-yl Carbamates †

**DOI:** 10.3390/molecules21091189

**Published:** 2016-09-07

**Authors:** Tomas Gonec, Sarka Pospisilova, Lucie Holanova, Josef Stranik, Aneta Cernikova, Valeria Pudelkova, Jiri Kos, Michal Oravec, Peter Kollar, Alois Cizek, Josef Jampilek

**Affiliations:** 1 Department of Chemical Drugs, Faculty of Pharmacy, University of Veterinary and Pharmaceutical Sciences, Palackeho 1, Brno 61242, Czech Republic; F15334@vfu.cz (J.S.); cernikova.aneta@gmail.com (A.C.); jurd@email.cz (J.K.); 2 Department of Infectious Diseases and Microbiology, Faculty of Veterinary Medicine, University of Veterinary and Pharmaceutical Sciences, Palackeho 1, Brno 61242, Czech Republic; sharka.pospisilova@gmail.com (S.P.); valeria.pudelkova@gmail.com (V.P.); cizeka@vfu.cz (A.C.); 3 Department of Human Pharmacology and Toxicology, Faculty of Pharmacy, University of Veterinary and Pharmaceutical Sciences, Palackeho 1, Brno 61242, Czech Republic; lucieholanova9@gmail.com (L.H.); kollarp@vfu.cz (P.K.); 4 Global Change Research Institute CAS, Belidla 986/4a, Brno 60300, Czech Republic; oravec.m@czechglobe.cz; 5 Department of Pharmaceutical Chemistry, Faculty of Pharmacy, Comenius University, Odbojarov 10, Bratislava 83232, Slovakia

**Keywords:** carbamates, hydroxynaphthalene-carboxamides, in vitro antibacterial activity, in vitro antimycobacterial activity, in vitro cytotoxicity assay, structure-activity relationships

## Abstract

Series of thirteen 1-[(2-chlorophenyl)carbamoyl]naphthalen-2-yl carbamates and thirteen 1-[(2-nitrophenyl)carbamoyl]naphthalen-2-yl carbamates with alkyl/cycloalkyl/arylalkyl chains were prepared and characterized. Primary in vitro screening of the synthesized compounds was performed against *Staphylococcus aureus*, two methicillin-resistant *S. aureus* strains, *Mycobacterium marinum*, and *M. kansasii.* 1-[(2-Chlorophenyl)carbamoyl]naphthalen-2-yl ethylcarbamate and 1-[(2-nitrophenyl)carbamoyl]naphthalen-2-yl ethylcarbamate showed antistaphylococcal (MICs = 42 µM against MRSA) and antimycobacterial (MICs = 21 µM) activity against the tested strains comparable with or higher than that of the standards ampicillin and isoniazid. In the case of bulkier carbamate tails (R > propyl/isopropyl), the activity was similar (MICs ca. 70 µM). Screening of the cytotoxicity of both of the most effective compounds was performed using THP-1 cells, and no significant lethal effect was observed (LD_50_ >30 µM). The structure-activity relationships are discussed.

## 1. Introduction

Infectious diseases represent an increasing worldwide threat. The number of untreatable diseases decreased after the 1950s due to the introduction of new antimicrobial agents. However, since the 1980s, morbidity has risen again. The increase in the number of new infections is caused by general immunosuppression, a significant increase in the number of diabetic or HIV-positive patients, and the development of resistance to commonly used drugs. The resistance of common pathogens to first-choice drugs increased by up to 100% during the last few decades. Moreover, the resistance of some strains to second- or third-choice drugs can be found. Development of cross-resistant and multidrug-resistant strains is a serious global problem. Selection of resistant pathogens is especially caused by irrational and unavailing application of anti-invasive agents in human, veterinary medicine, and in agriculture [1,2,3,4]. Resistance may complicate the treatment of infections regardless of how mild these infections were at the early stage [5,6]. This increasing resistance highlights the urgency of designing new effective anti-invasive drugs and developing strategies focused on overcoming drug resistance [5,6,7,8,9].

The discovery of salicylanilides dates back to early phenol applications; currently, salicylanilides are well-known organic compounds exhibiting a broad spectrum of biological activities such as anthelmintic, antibacterial, antimycobacterial, and antiviral, among others. Salicylanilides have been described to affect a wide range of targets, although the appropriate mechanism of action responsible for overall biological activities of these compounds has not been proposed so far. Thus, salicylanilides seem to be promising candidates for antibacterial agents, which could be a solution to the resistance challenges [10,11,12,13,14,15,16,17].

In addition, the presence of an amide (–CONH–) or carbamate (–OCONH–) group with a hydrophobic residue in its close vicinity is characteristic not only of a number of clinically used drugs [18], but also applied pesticides [19]. These moieties are important functional groups that are able, due to their electron properties, to interact and bind with a number of enzymes/receptors and, by means of these target sites, affect the biological response. The properties of the amide and the carbamate moieties can easily be modified by various substitutions [20,21]. Therefore, the reason for the widespread occurrence of amides and carbamates among new biologically active compounds is obvious [22,23,24,25,26,27,28,29,30,31,32,33,34,35,36].

1-[(2-Substituted phenyl)carbamoyl]naphthalen-2-yl carbamates, described in the present work, can be considered as cyclic analogues of salicylanilides that have expressed promising results as potential antibacterial and antimycobacterial agents ([14,15,16,17], and refs. therein). Pattern compounds of these carbamates–*N*-(2-chlorophenyl)-2-hydroxynaphthalene-1-carboxamide (**1**) and *N*-(2-nitrophenyl)-2-hydroxynaphthalene-1-carboxamide (**2**) showed antimycobacterial or antibacterial activity with insignificant cytotoxicity on human cells in the previous screening [30]; therefore, the aim of this contribution was to describe the preparation of various alkyl, cycloalkyl, and arylalkyl carbamates and the investigation of their antimicrobial activity.

## 2. Results and Discussion

### 2.1. Chemistry

**Scheme 1:**
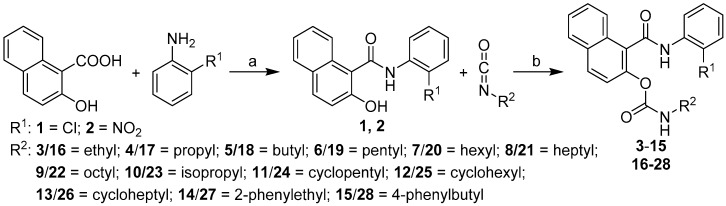
Synthesis of 1-[(2-chlorophenyl)carbamoyl]naphthalen-2-yl carbamates **3**–**15** and 1-[(2-nitrophenyl)carbamoyl]naphthalen-2-yl carbamates **16**–**28**. *Reagents and conditions*: (a) PCl_3_, chlorobenzene, MW; (b) TEA, acetonitrile, ambient temperature.

All the studied compounds were prepared according to Scheme 1. In the first step, *N*-(2-chlorophenyl)-2-hydroxynaphthalene-1-carboxamide (**1**) and *N*-(2-nitrophenyl)-2-hydroxy-naphthalene-1-carboxamide (**2**) were synthesized by the microwave-assisted method [30]. In the second step, a modified method using triethylamine for activation of the phenolic group was used [24]. The addition of activated compounds **1** and **2** to appropriate alkyl, cycloalkyl, and arylalkyl carbamates yielded a series of thirteen 1-[(2-chlorophenyl)carbamoyl]naphthalen-2-yl carbamates **3**–**15** and thirteen 1-[(2-nitrophenyl)carbamoyl]naphthalen-2-yl carbamates **16**–**28**.

In a number of studies examining the biological activity of potential drugs, the relationship between lipophilicity or other descriptors and their potency have been investigated. In the current investigation, the calculated lipophilicity (log *P*) of the compounds as well as the electronic parameters and molar volume of R^2^ substituents (see Table 1) were used to determine if these factors play a role in their biological activity. All the predicted molecular descriptors were calculated using the ACD/Percepta ver. 2012 program, see Table 1. The lipophilicity, expressed as log *P* values, of the chlorine substituted compounds **1**, **3**–**15** was higher (ranged from 3.94 to 5.75) than that of the nitro substituted derivatives **2**, **16**–**28** (ranged from 3.58 to 5.55). Lipophilicity increases with lengthening of the alkyl tail. Isopropyl showed a lower lipophilicity value than propyl. Significantly lower lipophilicity values were calculated for the cycloalkyl derivatives compared with their *N*-alkyl isomers. For individual R^2^ substituents, alkyl/cycloalkyl/arylalkyl tails of the discussed compounds, and also electronic properties expressed as electronic constants σ* were predicted; they ranged from −0.25 to 0.08. The electronic parameters (expressed as Hammett’s σ parameters) of the 2-Cl moiety (compound **1**) and the 2-NO_2_ moiety (compound **2**) were 0.22 and 0.77, respectively. Molar volume MV [cm^3^], a parameter representing the bulk of R^2^ substituents (i.e., tail length/branching) of each compound, was also calculated for the hydrophobic tail.

### 2.2. In Vitro Antibacterial Susceptibility Testing

The in vitro antibacterial activity of the discussed compounds was evaluated against two clinical isolates of methicillin-resistant *Staphylococcus aureus* (MRSA) and *S. aureus* ATCC 29213 as a reference and quality control strain. All the compounds showed only moderate or negligible activity, except ethylcarbamates **3** (R^1^ = Cl) and **16** (R^1^ = NO_2_). The activity of both ethylcarbamates **3** and **16** was comparable with that of the standard ampicillin; furthermore, the effect of compound **16** against *S. aureus* was ca. 4-fold higher in comparison with that of the chloro derivative **3**. In addition, nitrated propylcarbamate **17** showed ca. 8-fold higher activity than the corresponding chlorinated compound **4**, and nitro-anilide **2** had ca. 8-fold higher effectivity against *S. aureus* than chloro-anilide **1**. The observation that anilides substituted by the nitro moiety on the anilide ring showed higher potency only against *S. aureus* and not against MRSA strains in comparison with chlorine substituted derivatives was also described by Pauk et al. [37]. It can be supposed that nitro derivatives may interact by hydrogen bonding with specific biological structures in *S. aureus* (that are not present in MRSA strains). These interactions are limited only for spatially small molecules, such as anilide and ethyl-, propyl-, and isopropylcarbamate, while carbamates with bulkier (long or branched) tails are not capable of these interactions. This fact was also confirmed by Zadrazilova et al., where short ethylcarbamates demonstrated higher bacteriostatic activity against various *Staphylococcus* strains than longer (C_9_–C_12_) alkylcarbamates [16]. Due to the moderate activity of the rest of the compounds, no thorough structure-activity relationships could be established. Nevertheless, in general, it can be stated that the activity is influenced by similar factors/parameters as those discussed below.

### 2.3. In Vitro Antimycobacterial Evaluation

The evaluation of the in vitro antimycobacterial activity of the compounds was performed against *Mycobacterium marinum* CAMP 5644 (MM) and *M. kansasii* DSM 44162 (MK), see Table 1. To lower risks and make manipulation in the laboratory easier, surrogate model pathogens for *M. tuberculosis* can be used in laboratory studies. *Mycobacterium marinum* is very closely related to *M. tuberculosis* and is the cause of tuberculosis-like infections in poikilothermic organisms, especially frogs and fish. *M. marinum* is a good model for study especially because of the lower risk for laboratory workers, genetic relatedness, and pathology similar to human tuberculosis [38]. However, because of *M. tuberculosis,* the pathogenic role of nontuberculous mycobacteria (NTM) in humans was underestimated for a long time [39]. *M. kansasii*, the most virulent of the NTM, causes nontuberculous mycobacterial lung infections that are very common nowadays and can be indistinguishable from tuberculosis [40]. Therefore, additionally *M. kansasii* was chosen as a model species for screening of prospective antimycobacterial drugs to control mycobacterial diseases. The activity of the compounds was expressed as the minimum inhibitory concentration (MIC) that is defined for mycobacteria as a 90% or greater (IC_90_) reduction of growth in comparison with the control [41].

**Figure 1:**
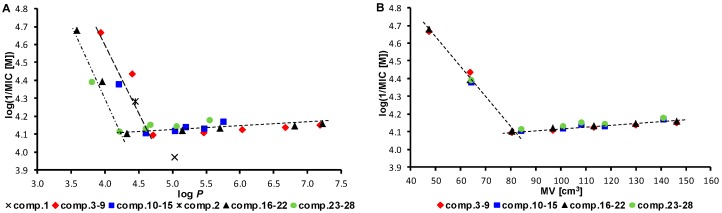
Dependence of the in vitro antimycobacterial activity against *M. kansasii* DSM 44162 log (1/MIC [M]) of the tested compounds on lipophilicity, expressed as log *P* (**A**) and bulkiness/molar volume (MV [cm^3^]) of individual R^2^ substituents (**B**).

**Figure 2:**
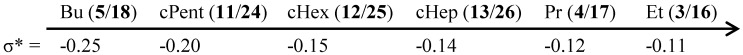
Increase of the general antimicrobial activity owing to the R^2^ substituent in dependence on the electronic properties are expressed as electronic constants σ*.

It can be stated that the potency of almost all discussed compounds is the same against both mycobacterial strains (see Table 1). Similarly, as mentioned above, ethylcarbamates **3** (R^1^ = Cl) and **16** (R^1^ = NO_2_) showed the highest antimycobacterial activity (MICs = 21 µM). In addition, propylcarbamates **4** (R^1^ = Cl) and **17** (R^2^ = NO_2_) expressed substantial activity (MICs ca. 40 µM). The dependences of the antimycobacterial activity expressed as log (1/MIC) on the lipophilicity (log *P*) of the compounds as well as on the bulkiness/molar volume (MV [cm^3^]) of individual alkyl/cycloalkyl/arylalkyl chains are illustrated in Figure 1A,B. Practically the same structure-activity relationships can be found for both series; therefore they are illustrated only for *M. kansasii*. The activity rapidly decreases with lipophilicity and with substituent bulkiness, increasing up to log *P* ca. 4.4 or MV ca. 80 cm^3^ (R^2^ = butyl or cyclopentyl), respectively, and then remains practically constant (or increases insignificantly) with increasing lipophilicity/bulkiness (see Figure 1A,B). For example, correlation factor r = 0.9919, n = 10 (compounds **3**–**5**, **10**, **11**, **16**–**18**, **23**, **24**) of the dependence of activity on the bulkiness of the R^2^ substituent can be calculated. It seems that the activity is also secondarily influenced by the electronic properties of R^2^ substituents expressed as electronic constants σ*, because it can be stated that the more the σ* values approximate −0.11 (i.e., the less electron donor properties are), the higher the activity is (see Figure 2).

In the previous studies [24,42], the most potent carbamates were substituted by a longer tail, which is connected with their surface activity. In this case, no dependence of antibacterial/antimycobacterial effects on surface activity was observed. As ethylcarbamates showed the highest activity in the discussed screening and propyl- and isopropyl-carbamates showed medium activity, it seems that steric requirements (spatially small molecules) play a significant role for a good biological effect. Similar facts were recently described, for example, by Kratky et al., where carbamates with spatially bulky tails expressed significantly lower activity than short-tail carbamates [43]. On the other hand, the blocking of the phenolic moiety by the carbamate group significantly increased the antimicrobial effect of these compounds. The mode of action (targets) of these compounds is questionable; however, due to the structural similarity with salicylanilides, interactions with vital enzymes, energy metabolism, as well as disturbing of the membrane architecture of prokaryotic cells may be supposed [10,11,13,14,15,16,17,32,33,36,44].

### 2.4. In Vitro Cytotoxicity Assay

The preliminary in vitro screening of the cytotoxicity of the compounds was performed using the human monocytic leukemia THP-1 cell line. The cytotoxicity was evaluated as the LD_50_ value (LD_50_—lethal dose to 50% of the cell population), see Table 1. A compound is considered cytotoxic when it demonstrates a toxic effect on cells at concentrations up to 10 μM [45], and the highest tested concentration that was used for the toxicity assay was 3-fold this value. Treatment with 30 μM compounds did not lead to lethal effects on the THP-1 cells. Based on these observations, it can be concluded that the most potent compounds **3** and **16** can be considered as promising agents for subsequent design of novel antibacterial and antimycobacterial agents.

## 3. Experimental Section

### 3.1. General Information

All reagents were purchased from Aldrich (Sigma-Aldrich, St. Louis, MO, USA), and Alfa (Alfa-Aesar, Ward Hill, MA, USA). TLC experiments were performed on alumina-backed silica gel 60 F254 plates (Merck, Darmstadt, Germany). The plates were illuminated under UV light (254 nm) and evaluated in iodine vapour. The melting points were determined on a Kofler hot-plate apparatus HMK (Franz Kustner Nacht KG, Dresden, Germany) and are uncorrected. Infrared (IR) spectra were recorded on a Smart MIRacle™ ATR ZnSe for Nicolet™ Impact 410 FT-IR spectrometer (Thermo Scientific, West Palm Beach, FL, USA). The spectra were obtained by the accumulation of 256 scans with a 2 cm^−1^ resolution in the region of 4000–650 cm^−1^. All ^1^H- and ^13^C-NMR spectra were recorded on a JEOL ECZR 400 MHz NMR spectrometer (400 MHz for ^1^H and 100 MHz for ^13^C, JEOL, Tokyo, Japan) in DMSO-*d*_6_. ^1^H and ^13^C chemical shifts (δ) are reported in ppm. High-resolution mass spectra were measured using a high-performance liquid chromatograph Dionex UltiMate^®^ 3000 (Thermo Scientific) coupled with a LTQ Orbitrap XL™ Hybrid Ion Trap-Orbitrap Fourier Transform Mass Spectrometer (Thermo Scientific) with injection into HESI II in the positive mode. The lipophilicity (log *P*) of the final compounds, the surface tension, and the molar volume of R substituents were predicted using ACD/Percepta ver. 2012 (Advanced Chemistry Development, Inc., Toronto, ON, Canada).

### 3.2. Synthesis

#### General Procedure for Synthesis of Carbamates **3**–**28**

The synthetic pathway and characterization of *N*-(2-chlorophenyl)-2-hydroxynaphthalene-1-carboxamide (**1**) and *N*-(2-nitrophenyl)-2-hydroxynaphthalene-1-carboxamide (**2**) were described recently by Gonec et al. [30]. Anilides **1** and **2** (1.0 mmol) and triethylamine (1.1 mmol) were dissolved in dry acetonitrile (10 mL). The solution of the appropriate alkyl isocyanate (1.2 mmol) in acetonitrile (5 mL) was added in four portions within 2 h, and the reacting mixture was stirred for 24 h at ambient temperature. The solvent was evaporated under reduced pressure, and the solid residue was washed with methanol and ethyl acetate to give pure product. All the studied compounds are presented in Table 1.

*1-[(2-Chlorophenyl)carbamoyl]naphthalen-2-yl ethylcarbamate* (**3**). Yield 33%; Mp 137–139 °C; IR (cm^−1^): 3264, 3214, 1718, 1675, 1540, 1523, 1510, 1481, 1435, 1300, 1256, 1229, 1006, 826, 741, 723, 688, 667; ^1^H-NMR (DMSO-*d*_6_) δ: 10.14 (s, 1H), 7.99–8.06 (m, 3H), 7.88 (t, *J* = 5.5 Hz, 1H), 7.82 (dd, *J* = 8.2 Hz, 1.3 Hz, 1H), 7.64 (ddd, *J* = 8.2 Hz, 6.9 Hz, 1.0 Hz, 1H), 7.55–7.59 (m, 2H), 7.43 (td, *J* = 7.7 Hz, 1.6 Hz, 1H), 7.42 (d, *J* = 9.0 Hz, 1H), 7.30 (td, *J* = 7.8 Hz, 1.3 Hz, 1H), 3.08–3.15 (m, 2H), 1.08 (t, *J* = 7.3 Hz, 3H); ^13^C-NMR (DMSO-*d*_6_), δ: 164.33, 153.97, 142.20, 134.65, 130.54, 130.31, 129.77, 129.60, 128.40, 128.04, 127.58, 127.30, 127.43, 127.28, 126.60, 125.78, 124.67, 122.62, 35.42, 14.83; HR-MS: for C_20_H_16_O_3_N_2_Cl [M + H]^+^ calculated 367.08440 *m*/*z*, found 367.08545 *m*/*z*.

*1-[(2-Chlorophenyl)carbamoyl]naphthalen-2-yl propylcarbamate* (**4**). Yield 26%; Mp 132–134 °C; IR (cm^−1^): 3259, 3224, 1716, 1659, 1546, 1526, 1510, 1484, 1441, 1298, 1259, 1232, 1060, 991, 809, 741, 694; ^1^H-NMR (DMSO-*d*_6_) δ: 10.10 (s, 1H), 7.99–8.05 (m, 3H), 7.89 (t, *J* = 5.7 Hz, 1H), 7.83 (dd, *J* = 8.2 Hz, 1.3 Hz, 1H), 7.64 (ddd, *J* = 8.2 Hz, 6.9 Hz, 1.0 Hz, 1H), 7.55–7.59 (m, 2H), 7.42 (td, *J* = 7.7 Hz, 1.6 Hz, 1H), 7.41 (d, *J* = 9.0 Hz, 1H), 7.30 (td, *J* = 7.8 Hz, 1.3 Hz, 1H), 3.04 (q, *J* = 6.4 Hz, 2H), 1.47 (sx, *J* = 7.2 Hz, 2H), 0.86 (t, *J* = 7.6 Hz, 3H); ^13^C-NMR (DMSO-*d*_6_), δ: 164.33, 154.20, 145.23, 134.67, 130.56, 130.31, 129.81, 129.59, 128.33, 128.04, 127.48, 127.42, 127.30, 127.28, 126.60, 125.78, 124.67, 122.62, 42.35, 22.45, 11.17; HR-MS: for C_21_H_18_O_3_N_2_Cl [M + H]^+^ calculated 381.10005 *m*/*z*, found 381.10123 *m*/*z.*

*1-[(2-Chlorophenyl)carbamoyl]naphthalen-2-yl butylcarbamate* (**5**). Yield 55%; Mp 156–159 °C; IR (cm^−1^): 3258, 3228, 1724, 1665, 1553, 1526, 1506, 1479, 1439, 1232, 1007, 818, 751; ^1^H-NMR (DMSO-*d*_6_) δ: 10.08 (s, 1H), 7.99–8.05 (m, 3H), 7.88 (t, *J* = 5.5 Hz, 1H), 7.85 (dd, *J* = 8.2 Hz, 1.3 Hz, 1H), 7.64 (ddd, *J* = 8.2 Hz, 6.9 Hz, 1.0 Hz, 1H), 7.55–7.59 (m, 2H), 7.42 (td, *J* = 7.7 Hz, 1.6 Hz, 1H), 7.41 (d, *J* = 9.0 Hz, 1H), 7.30 (td, *J* = 7.8 Hz, 1.3 Hz, 1H), 3.08 (q, *J* = 6.4 Hz, 2H), 1.44 (qi, *J* = 7.2 Hz, 2H), 1.28 (sx, *J* = 7.3 Hz, 2H), 0.85 (t, *J* = 7.3 Hz, 3H); ^13^C-NMR (DMSO-*d*_6_), δ: 164.31, 154.19, 145.23, 134.67, 130.57, 130.31, 129.84, 129.60, 128.20, 128.05, 127.41, 127.32, 127.31, 127.22, 126.59, 125.79, 124.65, 122.60, 40.26, 31.32, 19.37, 13.63; HR-MS: for C_22_H_20_O_3_N_2_Cl [M + H]^+^ calculated 395.11570 *m*/*z*, found 395.11639 *m*/*z.*

*1-[(2-Chlorophenyl)carbamoyl]naphthalen-2-yl pentylcarbamate* (**6**). Yield 36%; Mp 137–138 °C; IR (cm^−1^): 3227, 3215, 1724, 1669, 1550, 1521, 1508, 1480, 1434, 1233, 1037, 997, 910, 817, 746, 686; ^1^H-NMR (DMSO-*d*_6_) δ: 10.08 (s, 1H), 7.99–8.05 (m, 3H), 7.88 (t, *J* = 5.5 Hz, 1H), 7.85 (dd, *J* = 8.2 Hz, 1.3 Hz, 1H), 7.64 (ddd, *J* = 8.2 Hz, 6.9 Hz, 1.0 Hz, 1H), 7.55–7.59 (m, 2H), 7.39–7.44 (m, 2H), 7.30 (td, *J* = 7.8 Hz, 1.3 Hz, 1H), 3.07 (q, *J* = 6.7 Hz, 2H), 1.45 (qi, *J* = 7.0 Hz, 2H), 1.24–1.28 (m, 4H), 0.84 (t, *J* = 7.0 Hz, 3H); ^13^C-NMR (DMSO-*d*_6_), δ: 164.39, 154.24, 145.26, 134.68, 130.62, 130.36, 129.90, 129.65, 128.27, 128.10, 127.46, 127.40, 127.37, 127.31, 126.64, 125.87, 124.70, 122.65, 40.59, 28.91, 28.42, 21.87, 13.92; HR-MS: for C_23_H_22_O_3_N_2_Cl [M + H]^+^ calculated 409.13135 *m*/*z*, found 409.13303 *m*/*z.*

*1-[(2-Chlorophenyl)carbamoyl]naphthalen-2-yl hexylcarbamate* (**7**). Yield 50%; Mp 103–106 °C; IR (cm^−1^): 2954, 2924, 1725, 1673, 1587, 1525, 1516, 1463, 1440, 1301, 1241, 1210, 1037, 995, 813, 756, 747, 684; ^1^H-NMR (DMSO-*d*_6_) δ: 10.08 (s, 1H), 7.99–8.05 (m, 3H), 7.88 (t, *J* = 5.5 Hz, 1H), 7.85 (dd, *J* = 8.2 Hz, 1.3 Hz, 1H), 7.64 (ddd, *J* = 8.2 Hz, 6.9 Hz, 1.0 Hz, 1H), 7.55–7.59 (m, 2H), 7.39–7.44 (m, 2H), 7.30 (td, *J* = 7.8 Hz, 1.3 Hz, 1H), 3.07 (q, *J* = 6.4 Hz, 2H), 1.45 (qi, *J* = 7.0 Hz, 2H), 1.20–1.31 (m, 6H), 0.85 (t, *J* = 6.9 Hz, 3H); ^13^C-NMR (DMSO-*d*_6_), δ: 164.39, 154.26, 145.26, 134.70, 130.63, 130.37, 129.93, 129.66, 128.25, 128.13, 127.46, 127.39, 127.36, 127.30, 126.66, 125.88, 124.71, 122.65, 40.64, 31.03, 29.21, 25.92, 22.07, 13.96; HR-MS: for C_24_H_24_O_3_N_2_Cl [M + H]^+^ calculated 423.14700 *m*/*z*, found 423.14767 *m*/*z.*

*1-[(2-Chlorophenyl)carbamoyl]naphthalen-2-yl heptylcarbamate* (**8**). Yield 66%; Mp 89–90 °C; IR (cm^−1^): 3279, 3227, 2956, 2933, 2923, 1720, 1662, 1538, 1520, 1479, 1463, 1438, 1296, 1266, 1253, 1234, 1192, 1060, 986, 911, 816, 750; ^1^H-NMR (DMSO-*d*_6_) δ: 10.08 (s, 1H), 7.99–8.05 (m, 3H), 7.88 (t, *J* = 5.5 Hz, 1H), 7.86 (dd, *J* = 8.2 Hz, 1.3 Hz, 1H), 7.64 (ddd, *J* = 8.2 Hz, 6.9 Hz, 1.0 Hz, 1H), 7.55–7.59 (m, 2H), 7.39–7.44 (m, 2H), 7.30 (td, *J* = 7.8 Hz, 1.3 Hz, 1H), 3.07 (q, *J* = 6.4 Hz, 2H), 1.45 (qi, *J* = 7.0 Hz, 2H), 1.20–1.29 (m, 8H), 0.85 (t, *J* = 6.9 Hz, 3H); ^13^C-NMR (DMSO-*d*_6_), δ: 164.42, 154.29, 145.28, 134.71, 130.65, 130.39, 129.96, 129.68, 128.27, 128.14, 127.48, 127.41, 127.39, 127.31, 126.67, 125.90, 124.73, 122.67, 40.65, 31.26, 29.26, 28.48, 26.22, 22.11, 14.02; HR-MS: for C_25_H_26_O_3_N_2_Cl [M + H]^+^ calculated 437.16265 *m*/*z*, found 437.16431 *m*/*z.*

*1-[(2-Chlorophenyl)carbamoyl]naphthalen-2-yl octylcarbamate* (**9**). Yield 52%; Mp 115–116 °C; IR (cm^−1^): 3297, 3286, 2924, 2851, 1724, 1673, 1584, 1548, 1540, 1509, 1467, 1441, 1434, 1299, 1246, 1215, 1160, 993, 827, 756, 747, 734, 677; ^1^H-NMR (DMSO-*d*_6_) δ: 10.08 (s, 1H), 7.99–8.05 (m, 3H), 7.88 (t, *J* = 5.5 Hz, 1H), 7.85 (dd, *J* = 8.2 Hz, 1.3 Hz, 1H), 7.64 (ddd, *J* = 8.2 Hz, 6.9 Hz, 1.0 Hz, 1H), 7.54–7.59 (m, 2H), 7.39–7.43 (m, 2H), 7.30 (td, *J* = 7.8 Hz, 1.3 Hz, 1H), 3.07 (q, *J* = 6.4 Hz, 2H), 1.45 (qi, *J* = 7.0 Hz, 2H),1.21–1.30 (m, 10H), 0.86 (t, *J* = 6.9 Hz, 3H); ^13^C-NMR (DMSO-*d_6_*), δ: 164.33, 154.19, 145.23, 134.68, 130.57, 130.33, 129.87, 129.60, 128.22, 128.07, 127.40, 127.35, 127.33, 127.22, 126.61, 125.81, 124.67, 122.60, 40.59, 31.25, 29.20, 28.73, 28.64, 26.21, 22.10, 13.96; HR-MS: for C_26_H_28_O_3_N_2_Cl [M + H]^+^ calculated 451.17830 *m*/*z*, found 451.18018 *m*/*z.*

*1-[(2-Chlorophenyl)carbamoyl]naphthalen-2-yl isoproylcarbamate* (**10**). Yield 39%; Mp 167–169 °C; IR (cm^−1^): 3249, 3216, 1971, 1716, 1668, 1581, 1544, 1530, 1510, 1481, 1462, 1439, 1324, 1300, 1256, 1234, 1066, 1022, 962, 910, 820, 742, 697; ^1^H-NMR (DMSO-*d*_6_) δ: 10.09 (s, 1H), 7.99–8.06 (m, 3H), 7.86 (t, *J* = 5.5 Hz, 1H), 7.84 (dd, *J* = 8.2 Hz, 1.3 Hz, 1H), 7.64 (ddd, *J* = 8.2 Hz, 6.9 Hz, 1.0 Hz, 1H), 7.56–7.59 (m, 2H), 7.43 (td, *J* = 7.7 Hz, 1.6 Hz, 1H), 7.41 (d, *J* = 9.0 Hz, 1H), 7.30 (td, *J* = 7.8 Hz, 1.3 Hz, 1H), 3.69 (sx, *J* = 6.6 Hz, 1H), 1.12 (d, *J* = 6.6 Hz, 6H); ^13^C-NMR (DMSO-*d*_6_), δ: 164.42, 153.41, 145.28, 134.70, 130.60, 130.37, 129.90, 129.65, 128.34, 128.10, 127.49, 127.48, 127.36, 127.35, 126.67, 125.85, 124.70, 122.71, 42.91, 22.40; HR-MS: for C_21_H_18_O_3_N_2_Cl [M + H]^+^ calculated 381.10005 *m*/*z*, found 381.10123 *m*/*z.*

*1-[(2-Chlorophenyl)carbamoyl]naphthalen-2-yl cyclopentylcarbamate* (**11**). Yield 60%; Mp 135–137 °C; IR (cm^−1^): 3224, 2951, 1713, 1659, 1544, 1525, 1509, 1485, 1467, 1440, 1290, 1256, 1233, 1151, 1060, 1052, 1013, 911, 815, 784, 740, 688, 680; ^1^H-NMR (DMSO-*d*_6_) δ: 10.09 (s, 1H), 7.99–8.05 (m, 3H), 7.93 (d, *J* = 6.9 Hz, 1H), 7.83 (dd, *J* = 8.2 Hz, 1.3 Hz, 1H), 7.64 (ddd, *J* = 8.2 Hz, 6.9 Hz, 1.0 Hz, 1H), 7.55–7.59 (m, 2H), 7.39–7.43 (m, 2H), 7.30 (td, *J* = 7.8 Hz, 1.3 Hz, 1H), 3.87 (sx, *J* = 5.9 Hz, 1H), 1.76–1.85 (m, 2H), 1.60–1.70 (m, 2H), 1.46–1.55 (m, 4H); ^13^C-NMR (DMSO-*d*_6_), δ: 164.39, 153.66, 145.26, 134.69, 130.58, 130.34, 129.84, 129.62, 128.44, 128.07, 127.56, 127.43, 127.33, 127.33, 126.69, 125.81, 124.88, 122.74, 52.49, 32.14, 23.91; HR-MS: for C_23_H_20_O_3_N_2_Cl [M + H]^+^ calculated 409.13135 *m*/*z*, found 409.13258 *m*/*z.*

*1-[(2-Chlorophenyl)carbamoyl]naphthalen-2-yl cyclohexylcarbamate* (**12**). Yield 34%; Mp 129–132 °C; IR (cm^−1^): 3245, 3218, 2931, 2854, 1717, 1664, 1544, 1525, 1510, 1479, 1463, 1436, 1317, 1295, 1272, 1254, 1229, 1150, 1061, 1027, 1016, 911, 816, 778, 747, 682; ^1^H-NMR (DMSO-*d*_6_) δ: 10.08 (s, 1H), 7.99–8.05 (m, 3H), 7.86 (d, *J* = 7.7 Hz, 1H), 7.84 (dd, *J* = 8.2 Hz, 1.3 Hz, 1H), 7.64 (ddd, *J* = 8.2 Hz, 6.9 Hz, 1.0 Hz, 1H), 7.55–7.59 (m, 2H), 7.39–7.45 (m, 2H), 7.30 (td, *J* = 7.8 Hz, 1.3 Hz, 1H), 3.36–3.44 (m, 1H), 1.77–1.87 (m, 2H), 1.66–1.72 (m, 2H), 1.53–1.58 (m, 1H), 1.21–1.29 (m, 4H), 1.07–1.15 (m, 1H); ^13^C-NMR (DMSO-*d*_6_), δ: 164.38, 153.39, 145.27, 134.69, 130.56, 130.35, 129.85, 129.61, 128.38, 128.07, 127.50, 127.43, 127.32, 127.31, 126.67, 125.80, 124.68, 122.68, 49.99, 32.50, 25.12, 24.60; HR-MS: for C_24_H_22_O_3_N_2_Cl [M + H]^+^ calculated 423.14700 *m*/*z*, found 423.14818 *m*/*z.*

*1-[(2-Chlorophenyl)carbamoyl]naphthalen-2-yl cycloheptylcarbamate* (**13**). Yield 60%; Mp 166–168 °C; IR (cm^−1^): 3295, 3204, 2919, 2855, 1732, 1708, 1656, 1582, 1544, 1521, 1506, 1475, 1441, 1429, 1342, 1291, 1244, 1217, 1205, 1160, 1039, 1008, 991, 824, 772, 746, 712, 688; ^1^H-NMR (DMSO-*d*_6_) δ: 10.04 (s, 1H), 7.99–8.05 (m, 3H), 7.90 (d, *J* = 8.2 Hz, 1H), 7.85 (dd, *J* = 8.2 Hz, 1.3 Hz, 1H), 7.64 (ddd, *J* = 8.2 Hz, 6.9 Hz, 1.0 Hz, 1H), 7.55–7.59 (m, 2H), 7.39–7.45 (m, 2H), 7.30 (td, *J* = 7.8 Hz, 1.3 Hz, 1H), 3.52–3.60 (m, 1H), 1.80–1.86 (m, 2H), 1.43–1.65 (m, 8H), 1.32–1.42 (m, 2H); ^13^C-NMR (DMSO-*d*_6_), δ: 164.36, 153.29, 145.29, 134.68, 130.57, 130.34, 129.87, 129.60, 128.24, 128.06, 127.43, 127.34, 127.32, 127.25, 126.66, 125.80, 124.67, 122.70, 52.17, 34.35, 27.82, 23.58; HR-MS: for C_25_H_24_O_3_N_2_Cl [M + H]^+^ calculated 437.16265 *m*/*z*, found 437.16342 *m*/*z.*

*1-[(2-Chlorophenyl)carbamoyl]naphthalen-2-yl (2-phenylethyl)carbamate* (**14**). Yield 38%; Mp 115–118 °C; IR (cm^−1^): 3316, 3221, 1731, 1661, 1583, 1538, 1511, 1467, 1443, 1307, 1248, 1229, 1221, 1200, 1162, 1059, 1031, 984, 817, 751, 739, 698; ^1^H-NMR (DMSO-*d*_6_) δ: 10.13 (s, 1H), 7.99–8.06 (m, 4H), 7.84 (dd, *J* = 8.1 Hz, 1.4 Hz, 1H), 7.64 (ddd, *J* = 8.2 Hz, 6.9 Hz, 1.0 Hz, 1H), 7.55–7.59 (m, 2H), 7.43 (td, *J* = 7.7 Hz, 1.6 Hz, 1H), 7.37 (d, *J* = 9.1 Hz, 1H), 7.26–7.32 (m, 4H), 7.21–7.23 (m, 2H), 3.28–3.34 (m, 2H), 2.78 (t, *J* = 6.4 Hz, 2H); ^13^C-NMR (DMSO-*d*_6_), δ: 164.33, 154.13, 145.16, 139.13, 134.67, 130.57, 130.32, 129.81, 129.63, 128.70, 128.35, 128.28, 128.07, 127.50, 127.47, 127.33, 127.27, 126.55, 126.14, 125.82, 124.69, 122.55, 42.21, 35.23; HR-MS: for C_26_H_20_O_3_N_2_Cl [M + H]^+^ calculated 445.13135, found 445.13208 *m*/*z.*

*1-[(2-Chlorophenyl)carbamoyl]naphthalen-2-yl (4-phenylbutyl)carbamate* (**15**). Yield 58%; Mp 132–134 °C; IR (cm^−1^): 3220, 3024, 2938, 1719,1652, 1582, 1520, 1505, 1478, 1462, 1431, 1290, 1256, 1232, 1060, 985, 822, 760, 745, 699; ^1^H-NMR (DMSO-*d*_6_) δ: 10.10 (s, 1H), 7.99–8.05 (m, 3H), 7.90 (t, *J* = 5.5 Hz, 1H), 7.83 (dd, *J* = 8.2 Hz, 1.3 Hz, 1H), 7.63 (ddd, *J* = 8.2 Hz, 6.9 Hz, 1.0 Hz, 1H), 7.54–7.59 (m, 2H), 7.36–7.43 (m, 3H), 7.24–7.31 (m, 3H), 7.15–7.19 (m, 2H), 3.10 (q, *J* = 6.0 Hz, 2H), 2.55 (t, *J* = 7.3 Hz, 2H), 1.59 (qi, *J* = 7.3 Hz, 2H), 1.48 (qi, *J* = 7.3 Hz, 2H); ^13^C-NMR (DMSO-*d*_6_), δ: 164.34, 154.19, 145.22, 142.09, 134.68, 130.57, 130.32, 129.83, 129.62, 128.31, 128.23, 128.22, 128.07, 127.44, 127.42, 127.32, 127.26, 126.61, 125.81, 125.64, 124.68, 122.62, 40.39, 34.80, 28.88, 28.19; HR-MS: for C_28_H_24_O_3_N_2_Cl [M + H]^+^ calculated 473.16265, found 473.16342 *m*/*z.*

*1-[(2-Nitrophenyl)carbamoyl]naphthalen-2-yl ethylcarbamate* (**16**). Yield 40%; Mp 157–159 °C; IR (cm^−1^): 3320, 3216, 2963, 2917, 2843, 1743, 1705, 1656, 1645, 1608, 1580, 1532, 1504, 1462, 1432, 1357, 1341, 1294, 1270, 1250, 1216, 1199, 1162, 1144, 1079, 1034, 993, 822, 791, 779, 732, 667; ^1^H-NMR (DMSO-*d*_6_) δ: 10.94 (s, 1H), 8.07 (d, *J* = 9.1 Hz, 1H), 8.01–8.05 (m, 3H), 7.71–7.80 (m, 3H), 7.65 (ddd, *J* = 8.2 Hz, 6.9 Hz, 1.4 Hz, 1H), 7.59 (ddd, *J* = 8.2 Hz, 6.9 Hz, 1.4 Hz, 1H), 7.41–7.48 (m, 2H), 3.04–3.11 (m, 2H), 1.03 (t, *J* = 7.1 Hz, 3H); ^13^C-NMR (DMSO-*d*_6_), δ: 164.12, 153.71, 145.56, 142.98, 133.89, 130.62, 130.51, 130.33, 130.26, 128.10, 127.42, 125.91, 125.82, 125.70, 124.96, 124.90, 124.43, 122.62, 35.39, 14.75; HR-MS: for C_20_H_16_O_5_N_3_ [M + H]^+^ calculated 378.10845, found 378.10934 *m*/*z.*

*1-[(2-Nitrophenyl)carbamoyl]naphthalen-2-yl propylcarbamate* (**17**). Yield 75%; Mp 153–155 °C; IR (cm^−1^): 3298, 3223, 2960, 2930, 2873, 1727, 1717, 1662, 1589, 1537, 1520, 1505, 1488, 1463, 1440, 1353, 1285, 1257, 1224, 1146, 1106, 1051, 996, 981, 914, 865, 818, 779, 731, 666; ^1^H-NMR (DMSO-*d*_6_) δ: 10.93 (s, 1H), 8.07 (d, *J* = 9.1 Hz, 1H), 8.01–8.05 (m, 3H), 7.72–7.82 (m, 3H), 7.65 (ddd, *J* = 8.2 Hz, 6.9 Hz, 1.4 Hz, 1H), 7.59 (ddd, *J* = 8.2 Hz, 6.9 Hz, 1.4 Hz, 1H), 7.41–7.48 (m, 2H), 3.00 (q, *J* = 6.5 Hz, 2H), 1.42 (sx, *J* = 7.1 Hz, 2H), 0.80 (t, *J* = 7.3 Hz, 3H); ^13^C-NMR (DMSO-*d*_6_), δ: 164.14, 153.94, 145.61, 142.89, 133.91, 130.69, 130.53, 130.33, 130.33, 128.11, 127.43, 125.93, 125.79, 125.72, 125.64, 124.91, 124.46, 122.62, 42.29, 22.37, 11.10; HR-MS: for C_21_H_18_O_5_N_3_ [M + H]^+^ calculated 392.12410, found 392.12521 *m*/*z.*

*1-[(2-Nitrophenyl)carbamoyl]naphthalen-2-yl butylcarbamate* (**18**). Yield 82%; Mp 171–174 °C; IR (cm^−1^): 3293, 3222, 2957, 2873, 1724, 1661, 1589, 1549, 1532, 1520, 1505, 1471, 1463, 1439, 1350, 1279, 1257, 1227, 1145, 1109, 1006, 914, 865, 818, 779, 769, 731, 680, 667; ^1^H-NMR (DMSO-*d*_6_) δ: 10.92 (s, 1H), 8.07 (d, *J* = 9.1 Hz, 1H), 8.01–8.05 (m, 3H), 7.73–7.80 (m, 3H), 7.65 (ddd, *J* = 8.2 Hz, 6.9 Hz, 1.4 Hz, 1H), 7.58 (ddd, *J* = 8.2 Hz, 6.9 Hz, 1.4 Hz, 1H), 7.45 (td, *J* = 8.2 Hz, 1.4 Hz, 1H), 7.38 (d, *J* = 9.1 Hz, 1H), 3.03 (q, *J* = 6.2 Hz, 2H), 1.38 (qi, *J* = 7.0 Hz, 2H), 1.23 (sx, *J* = 7.0 Hz, 2H), 0.81 (t, *J* = 7.1 Hz, 3H); ^13^C-NMR (DMSO-*d*_6_), δ: 164.13, 153.91, 145.58, 142.77, 133.91, 130.74, 130.52, 130.32, 130.31, 128.10, 127.41, 125.91, 125.74, 125.73, 125.56, 124.91, 124.44, 122.59, 40.18, 31.23, 19.27, 13.58; HR-MS: for C_22_H_20_O_5_N_3_ [M + H]^+^ calculated 406.13975, found 406.14081 *m*/*z.*

*1-[(2-Nitrophenyl)carbamoyl]naphthalen-2-yl pentylcarbamate* (**19**). Yield 70%; Mp 141–143 °C; IR (cm^−1^): 3298, 3226, 2935, 2874, 1723, 1667, 1588, 1548, 1532, 1519, 1505, 1485, 1475, 1436, 1347, 1322, 1284, 1253, 1226, 1142, 1108, 1037, 1025, 996, 913, 866, 819, 778, 767, 730, 680, 660; ^1^H-NMR (DMSO-*d*_6_) δ: 10.93 (s, 1H), 8.07 (d, *J* = 9.1 Hz, 1H), 8.01–8.05 (m, 3H), 7.75–7.81 (m, 3H), 7.65 (ddd, *J* = 8.2 Hz, 6.9 Hz, 1.4 Hz, 1H), 7.58 (ddd, *J* = 8.2 Hz, 6.9 Hz, 1.4 Hz, 1H), 7.45 (td, *J* = 8.2 Hz, 1.4 Hz, 1H), 7.41 (d, *J* = 9.1 Hz, 1H), 3.02 (q, *J* = 6.4 Hz, 2H), 1.40 (qi, *J* = 7.0 Hz, 2H), 1.09–1.33 (m, 4H), 0.82 (t, *J* = 6.2 Hz, 3H); ^13^C-NMR (DMSO-*d*_6_), δ: 164.12, 153.89, 145.58, 142.80, 133.88, 130.72, 130.51, 130.31, 130.30, 128.10, 127.42, 125.91, 125.74, 125.72, 125.58, 124.91, 124.44, 122.61, 40.47, 28.77, 28.29, 21.76, 13.83; HR-MS: for C_23_H_22_O_5_N_3_ [M + H]^+^ calculated 420.15540, found 420.15643 *m*/*z.*

*1-[(2-Nitrophenyl)carbamoyl]naphthalen-2-yl hexylcarbamate* (**20**). Yield 58%; Mp 122–123 °C; IR (cm^−1^): 3332, 3272, 2929, 2868, 1709, 1665, 1609, 1589, 1541, 1514, 1472, 1443, 1352, 1295, 1250, 1215, 1205, 1158, 1148, 1113, 1046, 993, 978, 911, 863, 825, 779, 761, 738, 697, 672; ^1^H-NMR (DMSO-*d*_6_) δ: 10.93 (s, 1H), 8.07 (d, *J* = 9.1 Hz, 1H), 8.01–8.05 (m, 3H), 7.75–7.81 (m, 3H), 7.65 (ddd, *J* = 8.2 Hz, 6.9 Hz, 1.4 Hz, 1H), 7.58 (ddd, *J* = 8.2 Hz, 6.9 Hz, 1.4 Hz, 1H), 7.45 (td, *J* = 8.2 Hz, 1.4 Hz, 1H), 7.41 (d, *J* = 9.1 Hz, 1H), 3.02 (q, *J* = 6.0 Hz, 2H), 1.38 (qi, *J* = 6.4 Hz, 2H), 1.18–1.25 (m, 6H), 0.83 (t, *J* = 6.6 Hz, 3H); ^13^C-NMR (DMSO-*d*_6_), δ: 164.65, 154.44, 146.08, 143.25, 134.42, 131.24, 131.04, 130.83, 130.82, 128.61, 127.94, 126.44, 126.23, 126.23, 126.06, 125.42, 124.94, 123.10, 41.03, 31.42, 29.56, 26.28, 22.49, 14.38; HR-MS: for C_24_H_24_O_5_N_3_ [M + H]^+^ calculated 434.17105, found 434.17236 *m*/*z.*

*1-[(2-Nitrophenyl)carbamoyl]naphthalen-2-yl heptylcarbamate* (**21**). Yield 46%; Mp 101–102 °C; IR (cm^−1^): 3333, 3265, 2957, 2925, 2852, 1709, 1665, 1652, 1609, 1588, 1541, 1511, 1472, 1464, 1441, 1350, 1294, 1250, 1215, 1205, 1148, 1045, 979, 911, 824, 779, 755, 737, 696, 667; ^1^H-NMR (DMSO-*d*_6_) δ: 10.93 (s, 1H), 8.07 (d, *J* = 9.1 Hz, 1H), 8.01–8.05 (m, 3H), 7.75–7.81 (m, 3H), 7.65 (ddd, *J* = 8.2 Hz, 6.9 Hz, 1.4 Hz, 1H), 7.58 (ddd, *J* = 8.2 Hz, 6.9 Hz, 1.4 Hz, 1H), 7.45 (td, *J* = 8.2 Hz, 1.4 Hz, 1H), 7.41 (d, *J* = 9.1 Hz, 1H), 3.02 (q, *J* = 6.3 Hz, 2H), 1.39 (qi, *J* = 6.4 Hz, 2H), 1.17–1.28 (m, 8H), 0.85 (t, *J* = 6.6 Hz, 3H); ^13^C-NMR (DMSO-*d*_6_), δ: 164.12, 153.89, 145.58, 142.77, 133.88, 130.74, 130.53, 130.31, 130.31, 128.10, 127.42, 125.91, 125.73, 125.72, 125.58, 124.90, 124.44, 122.61, 40.50, 31.16, 29.11, 28.36, 26.07, 22.04, 13.93; HR-MS: for C_25_H_26_O_5_N_3_ [M + H]^+^ calculated 448.18670, found 448.18848 *m*/*z.*

*1-[(2-Nitrophenyl)carbamoyl]naphthalen-2-yl octylcarbamate* (**22**). Yield 37%; Mp 92–94 °C; IR (cm^−1^): 3315, 3230, 2924, 2850, 1704, 1653, 1589, 1528, 1508, 1485, 1463, 1435, 1360, 1293, 1271, 1251, 1219, 915, 834, 780, 759, 736, 701, 667; ^1^H-NMR (DMSO-*d*_6_) δ: 10.93 (s, 1H), 8.07 (d, *J* = 9.1 Hz, 1H), 8.01–8.05 (m, 3H), 7.75–7.81 (m, 3H), 7.65 (ddd, *J* = 8.2 Hz, 6.9 Hz, 1.4 Hz, 1H), 7.59 (ddd, *J* = 8.2 Hz, 6.9 Hz, 1.4 Hz, 1H), 7.46 (td, *J* = 8.2 Hz, 1.4 Hz, 1H), 7.41 (d, *J* = 9.1 Hz, 1H), 3.03 (q, *J* = 5.5 Hz, 2H), 1.39 (qi, *J* = 6.0 Hz, 2H), 1.18–1.29 (m, 10H), 0.86 (t, *J* = 6.4 Hz, 3H); ^13^C-NMR (DMSO-*d*_6_), δ: 164.12, 153.89, 145.58, 142.79, 133.86, 130.72, 130.51, 130.32, 130.31, 128.10, 127.42, 125.91, 125.75, 125.72, 125.58, 124.90, 124.44, 122.59, 40.50, 31.22, 29.09, 28.67, 28.59, 26.12, 22.07, 13.95; HR-MS: for C_26_H_28_O_5_N_3_ [M + H]^+^ calculated 462.20235, found 462.20410 *m*/*z.*

*1-[(2-Nitrophenyl)carbamoyl]naphthalen-2-yl isoproylcarbamate* (**23**). Yield 69%; Mp 172–176 °C; IR (cm^−1^): 3355, 3320, 2974, 1738, 1665, 1586, 1532, 1505, 1489, 1440, 1362, 1245, 1213, 1173, 1149, 1056, 1018, 953, 924, 826, 778, 763, 732; ^1^H-NMR (DMSO-*d*_6_) δ: 10.93 (s, 1H), 8.07 (d, *J* = 9.1 Hz, 1H), 8.01–8.05 (m, 3H), 7.75–7.80 (m, 3H), 7.66 (ddd, *J* = 8.2 Hz, 6.9 Hz, 1.4 Hz, 1H), 7.59 (ddd, *J* = 8.2 Hz, 6.9 Hz, 1.4 Hz, 1H), 7.46 (td, *J* = 8.2 Hz, 1.4 Hz, 1H), 7.42 (d, *J* = 9.1 Hz, 1H), 3.65 (sx, *J* = 6.4 Hz, 2H), 1.07 (d, *J* = 6.4 Hz, 6H); ^13^C-NMR (DMSO-*d*_6_), δ: 164.18, 153.10, 145.61, 142.94, 133.92, 130.69, 130.54, 130.37, 130.33, 128.14, 127.46, 125.96, 125.85, 125.80, 125.72, 124.94, 124.46, 122.71, 42.86, 22.32; HR-MS: for C_21_H_18_O_5_N_3_ [M + H]^+^ calculated 394.13975, found 394.14058 *m*/*z.*

*1-[(2-Nitrophenyl)carbamoyl]naphthalen-2-yl cyclopentylcarbamate* (**24**). Yield 28%; Mp 138–142 °C; IR (cm^−1^): 3295, 3219, 2960, 1719, 1661, 1588, 1519, 1487, 1438, 1351, 1283, 1255, 1222, 1144, 1078, 998, 822, 779, 732, 667; ^1^H-NMR (DMSO-*d*_6_) δ: 10.92 (s, 1H), 8.07 (d, *J* = 9.1 Hz, 1H), 8.01–8.05 (m, 3H), 7.84 (d, *J* = 6.9 Hz, 1H), 7.72–7.79 (m, 2H), 7.65 (ddd, *J* = 8.2 Hz, 6.9 Hz, 1.4 Hz, 1H), 7.59 (ddd, *J* = 8.2 Hz, 6.9 Hz, 1.4 Hz, 1H), 7.46 (td, *J* = 8.2 Hz, 1.4 Hz, 1H), 7.42 (d, *J* = 9.1 Hz, 1H), 3.83 (sx, *J* = 5.5 Hz, 1H), 1.72–1.80 (m, 2H), 1.56–1.63 (m, 2H), 1.42–1.49 (m, 4H); ^13^C-NMR (DMSO-*d*_6_), δ: 164.18, 153.41, 145.64, 142.94, 133.92, 130.69, 130.55, 130.36, 130.34, 128.14, 127.46, 125.97, 125.86, 125.80, 125.72, 124.95, 124.45, 122.73, 52.47, 32.06, 23.29; HR-MS: for C_23_H_20_O_5_N_3_ [M + H]^+^ calculated 420.15540, found 420.15620 *m*/*z.*

*1-[(2-Nitrophenyl)carbamoyl]naphthalen-2-yl cyclohexylcarbamate* (**25**). Yield 54%; Mp 140–143 °C; IR (cm^−1^): 3328, 3297, 2937, 2911, 2858, 1705, 1662, 1589, 1536, 1514, 1505, 1472, 1440, 1354, 1312, 1292, 1209, 1146, 1049, 1014, 823, 779, 761, 733, 697; ^1^H-NMR (DMSO-*d*_6_) δ: 10.91 (s, 1H), 8.07 (d, *J* = 9.1 Hz, 1H), 8.01–8.05 (m, 3H), 7.72–7.79 (m, 3H), 7.65 (ddd, *J* = 8.2 Hz, 6.9 Hz, 1.4 Hz, 1H), 7.58 (ddd, *J* = 8.2 Hz, 6.9 Hz, 1.4 Hz, 1H), 7.45 (td, *J* = 8.2 Hz, 1.4 Hz, 1H), 7.41 (d, *J* = 9.1 Hz, 1H), 3.26–3.33 (m, 1H), 1.71–1.76 (m, 2H), 1.63–1.68 (m, 2H), 1.51–1.56 (m, 1H), 1.02–1.27 (m, 5H); ^13^C-NMR (DMSO-*d*_6_), δ: 164.17, 153.14, 145.65, 142.90, 133.91, 130.70, 130.53, 130.36, 130.35, 128.13, 127.46, 125.96, 125.82, 125.79, 125.69, 124.93, 124.45, 122.67, 49.96, 32.39, 25.10, 24.56; HR-MS: for C_24_H_22_O_5_N_3_ [M + H]^+^ calculated 434.17105, found 434.17208 *m*/*z.*

*1-[(2-Nitrophenyl)carbamoyl]naphthalen-2-yl cycloheptylcarbamate* (**26**). Yield 58%; Mp 142–145 °C; IR (cm^−1^): 3324, 2912, 2855, 1703, 1662, 1588, 1528, 1505, 1472, 1440, 1353, 1291, 1245, 1218, 1203, 1141, 1041, 997, 909, 823, 779, 758, 737, 697; ^1^H-NMR (DMSO-*d*_6_) δ: 10.91 (s, 1H), 8.06 (d, *J* = 9.1 Hz, 1H), 8.01–8.05 (m, 3H), 7.81 (d, *J* = 8.2 Hz, 1H), 7.72–7.78 (m, 2H), 7.65 (ddd, *J* = 8.2 Hz, 6.9 Hz, 1.4 Hz, 1H), 7.58 (ddd, *J* = 8.2 Hz, 6.9 Hz, 1.4 Hz, 1H), 7.45 (td, *J* = 8.2 Hz, 1.4 Hz, 1H), 7.41 (d, *J* = 9.1 Hz, 1H), 3.46–3.54 (m, 1H), 1.73–1.80 (m, 2H), 1.39–1.60 (m, 8H), 1.29–1.38 (m, 2H); ^13^C-NMR (DMSO-*d*_6_), δ: 164.18, 153.01, 145.67, 142.82, 133.92, 130.73, 130.54, 130.35, 130.35, 128.13, 127.45, 125.95, 125.80, 125.80, 125.63, 124.93, 124.45, 122.70, 52.13, 34.26, 27.80, 23.54; HR-MS: for C_25_H_24_O_5_N_3_ [M + H]^+^ calculated 448.18670, found 448.18784 *m*/*z.*

*1-[(2-Nitrophenyl)carbamoyl]naphthalen-2-yl (2-phenylethyl)carbamate* (**27**). Yield 32%; Mp 127–130 °C; IR (cm^−1^): 3336, 1742, 1708, 1683, 1608, 1578, 1535, 1510, 1495, 1434, 1398, 1334, 1270, 1211, 1144, 1130, 967, 824, 758, 743, 698; ^1^H-NMR (DMSO-*d*_6_) δ: 10.96 (s, 1H), 8.07 (d, *J* = 9.1 Hz, 1H), 8.01–8.05 (m, 3H), 7.91 (t, *J* = 5.7 Hz, 1H), 7.71–7.79 (m, 2H), 7.65 (ddd, *J* = 8.2 Hz, 6.9 Hz, 1.4 Hz, 1H), 7.59 (ddd, *J* = 8.2 Hz, 6.9 Hz, 1.4 Hz, 1H), 7.46 (td, *J* = 8.2 Hz, 1.4 Hz, 1H), 7.38 (d, *J* = 9.1 Hz, 1H), 7.17–7.26 (m, 5H), 3.26 (q, *J* = 6.4 Hz, 2H), 2.73 (t, *J* = 7.3 Hz, 2H); ^13^C-NMR (DMSO-*d*_6_), δ: 164.10, 153.87, 145.54, 143.01, 139.13, 133.94, 130.63, 130.54, 130.35, 130.31, 128.88, 128.72, 128.31, 128.14, 127.46, 126.10, 125.98, 125.82, 123.69, 124.94, 124.48, 122.58, 42.16, 35.11; HR-MS: for C_26_H_20_O_5_N_3_ [M + H]^+^ calculated 456.15540, found 456.15712 *m*/*z.*

*1-[(2-Nitrophenyl)carbamoyl]naphthalen-2-yl (4-phenylbutyl)carbamate* (**28**). Yield 47%; Mp 109–110 °C; IR (cm^−1^): 3351, 3233, 2934, 1714, 1677, 1593, 1519, 1505, 1482, 1458, 1435, 1352, 1287, 1244, 1222, 1051, 1007, 816, 779, 752, 731, 967; ^1^H-NMR (DMSO-*d*_6_) δ: 10.94 (s, 1H), 8.07 (d, *J* = 8.7 Hz, 1H), 8.00–8.05 (m, 3H), 7.82 (t, *J* = 5.7 Hz, 1H), 7.70–7.72 (m, 2H), 7.65 (ddd, *J* = 8.2 Hz, 6.9 Hz, 1.4 Hz, 1H), 7.58 (ddd, *J* = 8.2 Hz, 6.9 Hz, 1.4 Hz, 1H), 7.43–7.46 (m, 1H), 7.41 (d, *J* = 9.1 Hz, 1H), 7.24–7.28 (m, 2H), 7.15–7.18 (m, 3H), 3.06 (q, *J* = 5.9 Hz, 2H), 2.52 (t, *J* = 7.3 Hz, 2H), 1.54 (qi, *J* = 7.3 Hz, 2H), 1.43 (qi, *J* = 7.3 Hz, 2H); ^13^C-NMR (DMSO-*d*_6_), δ: 164.13, 153.93, 145.59, 142.96, 142.09, 133.88, 130.65, 130.54, 130.35, 130.32, 128.30, 128.22, 128.13, 127.45, 125.95, 125.80, 125.72, 125.64, 125.64, 124.93, 124.47, 122.63, 40.32, 34.77, 28.80, 28.11; HR-MS: for C_28_H_24_O_5_N_3_ [M + H]^+^ calculated 484.18670, found 484.18801 *m*/*z.*

### 3.3. In Vitro Antibacterial Susceptibility Testing

The synthesized compounds were evaluated for in vitro antibacterial activity against representatives of multidrug-resistant bacteria and clinical isolates of methicillin-resistant *Staphylococcus aureus* (MRSA) SA 630 and SA 3202, that were obtained from the National Institute of Public Health (Prague, Czech Republic). *Staphylococcus aureus* ATCC 29213 was used as a reference and quality control strain. Ampicillin (Sigma-Aldrich) was used as the standard. Prior to testing, each strain was passaged onto nutrient agar (Oxoid, Hampshire, UK) with 5% of bovine blood, and bacterial inocula were prepared by suspending a small portion of bacterial colony in sterile phosphate buffered saline (pH 7.2–7.3). The cell density was adjusted to 0.5 McFarland units using a densitometer (Densi-La-Meter, LIAP, Riga, Latvia). The final inoculum was made to a 1:20 dilution of the suspension with the Mueller-Hinton broth (MH broth). The compounds were dissolved in DMSO (Sigma), and the final concentration of DMSO in the MH broth (Oxoid) did not exceed 2.5% of the total solution composition. The final concentrations of the evaluated compounds ranged from 256 μg/mL to 0.008 μg/mL. The broth dilution micro-method, modified according to NCCLS (National Committee for Clinical Laboratory Standards) guidelines [46,47] in MH broth, was used to determine the minimum inhibitory concentration (MIC). Drug-free controls, sterility controls, and controls consisting of MH broth and DMSO alone were included. The determination of results was performed visually after 24 h of static incubation in the darkness at 37 °C in an aerobic atmosphere. The MICs were defined as the lowest concentration of the compound at which no visible bacterial growth was observed. The results are summarized in Table 1.

### 3.4. In Vitro Antimycobacterial Evaluation

The evaluation of the in vitro antimycobacterial activity of the compounds was performed against *Mycobacterium marinum* CAMP 5644 and *M. kansasii* DSM 44162. The broth dilution micro-method in Middlebrook 7H9 medium (Difco, Lawrence, KS, USA) supplemented with ADC Enrichment (Becton, Dickinson & Comp., Franklin Lakes, NJ, USA) was used to determine the minimum inhibitory concentration (MIC), as previously described [48]. The compounds were dissolved in DMSO (Sigma-Aldrich), and the final concentration of DMSO did not exceed 2.5% of the total solution composition. The final concentrations of the evaluated compounds, ranging from 256 μg/mL to 0.125 μg/mL, were obtained by twofold serial dilution of the stock solution in a microtiter plate with sterile medium. Bacterial inocula were prepared by transferring colonies from the culture to sterile water. The cell density was adjusted to 0.5 McFarland units using a densitometer (Densi-La-Meter, LIAP, Riga, Latvia). The final inoculum was made by 1:1000 dilution of the suspension with sterile water. Drug-free controls, sterility controls, and controls consisting of the medium and DMSO alone were included. The determination of results was performed visually after 7 days of static incubation in the darkness at 37 °C in an aerobic atmosphere for *M. kansasii* and after 21 days of static incubation in the darkness at 28 °C in an aerobic atmosphere for *M. marinum*. The minimum inhibitory concentration (MIC) was defined as the lowest concentration of the compound at which no visible bacterial growth was observed. The MIC value is routinely and widely used in bacterial assays and is a standard detection limit according to the Clinical and Laboratory Standards Institute (CLSI) [49]. Isoniazid (Sigma-Aldrich) was used as the reference antibacterial drug. The results are summarized in Table 1.

### 3.5. In Vitro Cytotoxicity Assay

Human monocytic leukemia THP-1 cells were obtained from the European Collection of Cell Cultures (ECACC, Salisbury, UK; Methods of characterization: DNA Fingerprinting (Multilocus probes) and isoenzyme analysis). These cells were routinely cultured in RPMI 1640 (Lonza, Verviers, Belgium) medium supplemented with 10% fetal bovine serum (FBS, Sigma-Aldrich), 2% l-glutamine, 1% penicillin, and streptomycin (Lonza, Verviers, Belgium) at 37 °C with 5% CO_2_. Cells were passaged at approximately 1-week intervals. Cells were routinely tested for the absence of mycoplasma (Hoechst 33258 staining method). The tested compounds were dissolved in DMSO (Sigma-Aldrich) and added in five increasing concentrations to the cell suspension in the culture medium. The maximum concentration of DMSO in the assays never exceeded 0.1%. Subsequently, the cells were incubated for 24 h at 37 °C with 5% CO_2_ at various compound concentrations ranging from 0.37 to 30 μmol/L in RPMI 1640 medium. Cell toxicity was determined using a Cytotoxicity Detection Kit^PLUS^ Lactate dehydrogenase (LDH) assay kit (Roche Diagnostics, Mannheim, Germany), and used according to the manufacturer’s instructions, as described previously [14,30,32,33]. For LDH assays, cells were seeded into 96-well plates (5 × 10^4^ cells·well^−1^ in 100 μL culture medium) in triplicate in serum-free RPMI 1640 medium, and measurements at 492 nm wavelength (Synergy 2 Multi-Mode Microplate Reader, BioTek, Winooski, VT, USA) were taken 24 h after the treatment with tested compounds. The median lethal dose values, LD_50_, were deduced through the production of a dose-response curve. All data were evaluated using GraphPad Prism 5.00 software (GraphPad Software, San Diego, CA, USA). The results are summarized in Table 1.

## 4. Conclusions

Series of thirteen 1-[(2-chlorophenyl)carbamoyl]naphthalen-2-yl carbamates and thirteen 1-[(2-nitrophenyl)carbamoyl]naphthalen-2-yl carbamates were prepared and subsequently characterized. All compounds were tested for their in vitro antimicrobial activity against *S. aureus*, two methicillin-resistant *S. aureus* strains, *M. marinum*, and *M. kansasii*. 1-[(2-Chlorophenyl)-carbamoyl]naphthalen-2-yl ethylcarbamate (**3**) and 1-[(2-nitrophenyl)carbamoyl]naphthalen-2-yl ethylcarbamate (**16**) showed MICs = 42 µM against all methicillin-resistant *S. aureus* strains. In addition, both compounds expressed MICs = 21 µM against *M. marinum* and *M. kansasii*. Propyl- (**4**, **17**) and isopropyl- (**10**, **23**) carbamates demonstrated medium antimicrobial activity, while bulkier-substituted carbamates showed only moderate or no activity, which was similar in both the chlorinated and nitrated series. Screening of the cytotoxicity of the most potent and discussed compounds, performed using the THP-1 cells, proved no significant lethal effect (LD_50_ >30 µM). The biological activity of the compounds is dependent on the alkyl substitution of carbamate nitrogen. Spatially small *N*-alkyl substituents are important for the activity. Antimicrobial activity rapidly decreases with chain prolongation or with a bulkier *N*-cycloalkyl, *N*-arylalkyl substituent.

## Figures and Tables

**Table 1:** Structures of the discussed anilides **1**, **2** and carbamates **3**–**28**; calculated values of log *P*, electronic constants σ*, and molar volume (MV [cm^3^]) of R^2^ substituents; in vitro antibacterial activity (MIC) of the compounds in comparison with the ampicillin (APC) standard; in vitro antimycobacterial activity (MIC) of the compounds in comparison with the isoniazid (INH) standard and in vitro cytotoxicity assay (LD_50_) of the chosen compounds.

Compd.	R^1^	R^2^	log *P* ^a^	MV_R2_^a^ [cm^3^]	σ*_R2_^a^	MIC [µM]					LD_50_ [µM]
						SA	MRSA SA 630	MRSA 3202	MM	MK	
**1**	Cl	−	5.03	–	–	215	860	860	107	107	>30
**3**	Cl	Et	3.94	47.29	−0.11	21.6	43.3	43.3	21.6	21.6	˃30
**4**	Cl	Pr	4.41	63.80	−0.12	83.5	83.5	83.5	41.7	36.5	˃30
**5**	Cl	Bu	4.71	80.31	−0.25	645	645	645	80.6	80.6	–
**6**	Cl	Pen	5.47	96.81	−0.23	623	623	623	77.8	77.8	–
**7**	Cl	Hex	6.03	113.32	−0.25	602	602	602	75.3	75.3	–
**8**	Cl	Hep	6.67	129.83	−0.23	583	583	583	72.9	72.9	–
**9**	Cl	Oct	7.19	146.33	−0.23	565	565	565	70.6	70.6	–
**10**	Cl	i-Pr	4.20	64.18	−0.19	83.5	167	167	41.7	41.7	˃30
**11**	Cl	c-Pent	4.60	84.10	−0.20	313	313	626	78.2	78.2	–
**12**	Cl	c-Hex	5.03	100.87	−0.15	303	605	303	75.6	75.6	–
**13**	Cl	c-Hep	5.46	117.56	−0.14	146	586	586	73.2	73.2	–
**14**	Cl	PhEt	5.19	108.00	0.08	288	575	575	71.9	71.9	–
**15**	Cl	PhBu	5.75	141.01	−0.21	271	541	541	67.6	67.6	–
**2**	NO_2_	−	4.45	–	–	26	104	52	104	51.9	>30
**16**	NO_2_	Et	3.58	47.29	−0.11	5.27	42.1	42.1	21.0	21.0	˃30
**17**	NO_2_	Pr	3.96	63.80	−0.12	10.1	81.3	81.3	40.6	40.6	˃30
**18**	NO_2_	Bu	4.32	80.31	−0.25	628	628	628	78.5	78.5	–
**19**	NO_2_	Pen	5.15	96.81	−0.23	607	607	607	75.9	75.9	–
**20**	NO_2_	Hex	5.71	113.32	−0.25	587	587	587	73.4	73.4	–
**21**	NO_2_	Hep	6.81	129.83	−0.23	569	569	569	71.1	71.1	–
**22**	NO_2_	Oct	7.22	146.33	−0.23	552	552	552	69.0	69.0	–
**23**	NO_2_	i-Pr	3.80	64.18	−0.19	40.6	162	162	40.6	40.6	˃30
**24**	NO_2_	c-Pent	4.21	84.10	−0.20	153	610	610	76.2	76.2	–
**25**	NO_2_	c-Hex	4.60	100.87	−0.15	74	591	591	73.8	73.8	–
**26**	NO_2_	c-Hep	5.05	117.56	−0.14	72	572	572	71.5	71.5	–
**27**	NO_2_	PhEt	4.67	108.00	0.08	281	562	562	70.2	70.2	–
**28**	NO_2_	PhBu	5.55	141.01	−0.21	132	529	529	66.1	66.1	–
**APC**	–		−	–	–	5.72	45.8	45.8	–	–	–
**INH**	–		−	–	–	–	–	–	467	29.2	–

^a^ calculated using ACD/Percepta ver. 2012 (Advanced Chemistry Development, Toronto, ON, Canada); SA = *S. aureus* ATCC 29213, MRSA = clinical isolates of methicillin-resistant *S. aureus* SA 630 and 3202 (National Institute of Public Health, Prague, Czech Republic); MM = *M. marinum* CAMP 5644, MK = *M. kansasii* DSM 44162.
